# Rheumatism, and a Plea for Higher Potencies

**Published:** 1888-07

**Authors:** Edward Rushmore

**Affiliations:** Plainfield, N. J.


### RHEUMATISM, AND A PLEA FOR THE HIGHER POTENCIES.

(Read before the Homœopathic Medical Society of New Jersey, May 1st, 1888.)

I was called one evening to a boy sick with acute rheumatism, then suffering excruciatingly from pain in the lower abdomen, left testicle, and left thigh ; he became delirious during the attacks, thinking his attendants were hurting him, and also saying "Give me something to tear." I told them to give him a newspaper, which he tore up quickly. The violent pains causing delirium and the desire to tear something seemed equally to call for Veratrum album, which I gave in the 200th potency with the most rapid and complete relief. At another time he suffered most painful urging to urinate, with erections and bloody urine, the attacks coming and ceasing suddenly. I had given Cantharides without relief, when my friend, Dr. Griffin, who saw the case with me, advised Belladonna, which I gave at once in the 50M potency with prompt and lasting relief.

On January 16th I was called to see Mrs. ——, who had been in bed three weeks with an attack of rheumatism in the right shoulder and arm, and had had old school treatment, which included hypodermics of Morphia. The rheumatism had been preceded by neuralgia over the right eye with fever for three weeks, for which she said she had Arsenic and much Quinine. She now has pains in the right shoulder and scapula, which extends down the arm. The pain is an aching, almost constant, and is worse from any motion even of the hand. But I found she could bear passive motion, yet wanted to keep the part still. Much thirst. No stool for three days. Temperature, 100. Pulse, 120. She received Bryonia$^{50m}$ (Skinner) in water, a teaspoonful every hour to four hours, according to severity of pain.

January 17th.—Less pain ; slept much ; bitter taste in morning. Pulse, 100. No medicine, as the case was clearly better.

January 18th.—Tongue cleaning; less thirst. Pulse, 90. No medicine, but has Bryonia to take if the pain returns. After that she improved, and I made no note of the case till February 6th, when I wrote: "Is improving. Bowels move occasionally with enema; she had desire for stool, but not strength enough to expel it. Tongue clean. Appetite good. She sits up all day and uses the arm much.

February 14th.—Skin dry; no sweat; mostly needs an enema for stool. Sulph.$^{cm}$, Fincke, one dose.

February 21st.—Has natural daily stools without enema. Has a restlessness of the legs coming right after supper and preventing sleep. I gave Platina$^{9c}$ (Fincke), one dose dry on the tongue.

March 5th.—The restlessness of the limbs, which was better, has returned again, preventing sleep. Platina$^{6m}$ (Fincke), one powder, was given and completed the cure.

Case III.—February 13th, 1888.—Mrs. M. Rheumatism in left shoulder beginning two days ago. She took Bell. yesterday, since which time she has had less pain and more sweat at night. The shoulder feels very heavy, and is the seat of an acute beating pain, worse from moving even the fingers. Headache; severe backache; tongue dry, with thin, yellow coating; taste bitter; no stool for two days. She received Bryonia$^{50m}$ (Skinner), three doses in water two hours apart. She was seen daily for some days, steadily improving, and getting only one more dose of Bryonia in the same potency, dry, when she complained on the 20th of ineffectual desire for stool, heaviness in the head, and want of appetite. These were removed by a dose of Sulphur $^{cm}$.

I have presented these cases as examples of improvement quickly following the use of remedies chosen by the rule of similarity of symptoms and given in an infinitesimal dose, and I think those of you who have seen much of the disease will agree with me that the results leave little to be desired. You will notice that one of these cases had been in bed three weeks under old school treatment, and that another was a recent attack. It, as well as the first patient mentioned, was also in bed.

I hope there is not now any need to plead before this Society that similarity of symptoms in drug effect and in disease is the basis of scientific prescribing, and such prescribing the only way of securing the beneficent action of natural law in the work of medicinal cure. But I would like to inquire, what is the ground of our belief in the truth of the homœopathic law? Does our belief rest on any speculative foundation? Has it been deduced from the principles of philosophy, or inferred from the postulates of logic? No; it rests on the sure foundation of demonstrated and even more demonstrable fact. Its perpetual defense is the daily repeated, daily verifiable experiment. Given the assertion of a truth and the means of its verification, with whom rests the responsibility of ignorance? When Dr. Gross said to the American Medical Association not many years ago, "Of the reputed virtues of Aconite as an antiphlogistic, we know nothing," he said it in the face of scientific testimony growing stronger through three-quarters of a century. But was Aconite any less active because of Dr. Gross' ignorance and neglect? How many homœopathic physicians are there who would not now have to say, "Of the reputed therapeutic powers of high potencies we know nothing"? Yet the high potencies go on working, curing, in spite of all ignorance and unbelief. Truth is constant, patient, enduring, beneficent, and secure. Hence she is alwaysat our side in loving ministry. In the literature of Homœopathy the evidences of the higher attenuations being often, if not always, really stronger, therapeutic potencies is yearly multiplying. I would therefore ask my medical brethren to consider whether they are doing their duty as healers, whether they are not neglecting the rights of the sick to whatever is best in medicine, in neglecting to put to the test of experiment the claims of the potencies as healing powers. The same method of proof or refutation is available in this case as in the question of the truth of the law of similars. Hahnemann challenged the opponents of Homœopathy to show by easy experiment, what ridicule or invective have never shown, that his doctrine was false. In how many a notable case, like that of Hering, did the attempted refutation turn against the refuter, and convince him against his prejudice by the clear logic of facts.

But do I hear it said that you have tried the higher potencies and that they don't work? Yes, I have heard it so said. Do you remember the case of Andral, who professed to try Homœopathy in Paris more than half a century ago, how without any knowledge of the homœopathic remedies he used them in certain cases and said they all failed? And how it was shown in a medical journal of the time that he made a wrong application of every one that he employed? We must not charge failure, therefore, when, the conditions of the experiment being disregarded, no experiment really is made. And the gentlemen who have personally told me the higher potencies were not satisfactory were by their own statement not accustomed to the use of the single remedy chosen according to homœopathic indications, and were therefore presumably ignorant, like Andral, of the first elements of a homœopathic prescription, and for this reason incapable of making a scientific experiment in the case. For to prescribe homœopathically it is necessary to know much more of the patient than is requisite to name his disease, much more of drugs than their established relations with nosology.

Gentlemen, the question of potency is not a question for partisan prejudice nor for speculative treatment; it is a question of simple science, susceptible of determination by the methods of science. I do not stand before you to denounce the use of lower potencies. Their effectiveness has been invulnerably established. But how? By repeated experiment. It is only by the same method that the higher have obtained with many a place of preference and of increased confidence. They who stand securely on the higher rounds of a ladder, know too well how the lower helped them, ever to despise the lower; but the wider horizon and clearer perspective found above forbid all desire to descend. And so the masters in our art have seen in the working of the higher potencies an enlarged horizon of therapeutic possibilities which they do not wish to lose. I want to say here how false is the belief, how mistaken the persons, who yet inculcate the belief that there is any schism in the homœopathic school on the ground of potencies, high or low. True, there is much difference, but difference in potency employed does not constitute difference in kind, nor hinder mutual sympathy, co-operation, and support. But the schism that *is*, the wearing of the fair name of Homœopathy and then dragging her colors in the dust under the specious pretense of doing *anything* to save the life of the patient, as if Homœopathy could not always do the *best*, and which seeks support and credence in ridiculing the infinitesimals of Homœopathy, as her bitterest enemies have done, this I repeat is the schism which is hated of her soul. Let us read what her greatest minister says of this and of his experience of the dose in a single sentence in his *Organon*: "Least, I say, since it stands and will stand as a homœopathic rule of cure, refutable by no experience in the world, that of the rightly chosen remedy the best dose is always the least, in one of the high potencies (thirtieth), as well for chronic as for acute diseases —a truth which is the invaluable property of pure Homœopathy, which, as long as allopathy (and not much less the modern mongrel sect, composed of allopathic and homœopathic treatment) yet continues to gnaw like a cancer upon the life of the sick, and to corrupt them with greater and greatest doses of medicine which will keep these false arts remote from the pure Homœopathy by an immeasurable gulf."

Returning now to the subject of potency, let me briefly revive the testimony of some of the leading experimenters in our school. Says Dr. Watzke in the *Austrian Journal:* "I am, alas!—I say, alas! for I would much rather have upheld the larger doses which accord with the current views—I am compelled to declare myself for the higher dilutions. The physiological experiments made with Natrum muriaticum, as well as the great majority of the clinical results obtained therewith, speak decisively and distinctly for these preparations." During the ten years from 1850 to 1859 inclusive one hundred and forty cases of pneumonia are reported as treated in the Leopoldstadt Hospital in Vienna, under the care of Drs. Wurmb and Eidherr. During the first three years fifty-five cases were treated with 30x potency; during the next three years thirty-one cases with the 6x potency, and during the last four years fifty-four cases with the 15x. Most careful and varied records were kept, with this result as to the duration of the sickness: The group treated with the sixth decimal, average duration, 19.5 days; group treated with 15x, average duration, 14.6 days; group treated with 30x, average duration, 11.3 days. We see in this most extensive, carefully recorded comparison, how the use of the higher potency was in both cases followed by a lessened duration of the disease.

Dr. Von Bœnninghausen, who even rivaled Hahnemann himself in therapeutic skill, "expressed a decided conviction of the superiority of the high potencies over the lower in both acute and chronic diseases."

Dr. Aegidi, "after a long course of experiment embracing the history of more than four thousand cases, announces a clear and unequivocal preference for the high potencies in both acute and chronic diseases."

And so I could go on adding name to name in the list of those who, in our own as well as in foreign lands, in our own as in times gone by, whose sufferings for Homœopathy have done so much to lighten our labors now, and whose names are her renown, who have made the needed experiment and become persuaded of this same truth.

I would close with the remarks of the veteran Bayard in his tribute to the memory of Constantine Hering: "I remember," he says, "on a certain occasion early in my practice I told Dr. Hering of my suffering. He asked me the remedy I had taken and seemed to think it well chosen. He then asked the dilution. I told him the third. 'Ah!' said he, 'you have stopped it, but perhaps not made a cure.' He shook his head and seemed much disappointed. He said no more, but he caused me to reflect that it might well be so, that I had thrown an obstacle before the diverted vital force—that I had stayed its forward movement by a shock that injured its reactive power, as a boulder thrown before a carriage wheel in motion stops it, but cripples the wheel."

EDWARD RUSHMORE, M. D.

PLAINFIELD, N. J.

